# Natural polymorphisms in the resistance associated sites of HCV-G1 NS5B domain and correlation with geographic origin of HCV isolates

**DOI:** 10.1186/s12985-018-1054-z

**Published:** 2018-09-18

**Authors:** Sabrina Bagaglio, Caterina Uberti-Foppa, Alessandro Olgiati, Emanuela Messina, Hamid Hasson, Camilla Ferri, Giulia Morsica

**Affiliations:** 1 Division of Infectious Diseases, Ospedale San Raffaele, Via Stamira d’Ancona 20, 20127 Milan, Italy; 2 Vita Salute University, Milan, Italy; 3 Pharmacy, Ospedale San Raffaele, Milan, Italy

**Keywords:** Anti-HCV direct acting antivirals (DAAs), Geographic distribution, HCV genotype, NS5b polymerase inhibitors, Resistance associated substitutions (RASs)

## Abstract

**Background:**

We evaluated the frequency of naturally occurring resistance associated substitutions (RASs) and their characteristic of polymorphic or non-polymorphic amino acid change to direct acting antivirals (DAAs) in NS5b HCV subtypes 1a and 1b according to different geographic origin of isolates.

**Methods:**

Using a public database we retrieved 738 worldwide NS5b sequences (for which was available the geographic origin) from HCV genotype (G)1 infected patients naive to DAAs. NS5b sequences clustering with G1a were more conserved in regard of RASs than G1b isolates, (14% vs 57% RASs, *P* < 0.0001).

**Results:**

In G1a, RASs were differently distributed between isolates from Europe (24%) and USA, (12%) *P* = 0.0186. In particular, 421 V associated with resistance to non-nucleoside inhibitor beclabuvir was polymorphic in Europe and USA, being detected in 24% and 11% of sequences, respectively, *P* = 0.0140.

In G1b, RASs were found in 45% of sequences from Europe, in 54% of isolates from USA and in 70% of sequences from Asia (*P* = 0.0051).

The 316 N polymorphism was detected in 54% of Asian isolates and at lower frequency, in 28% of isolates from USA and in 20% of European sequences (*P* < 0.0001).

**Conclusions:**

In conclusion, a higher prevalence of RASs in G1b respect to G1a was found and a geographical distribution of RASs and polymorphic aa changes was observed in G1a as well in G1b.

The clinical and therapeutic impact of the geographic distribution of RASs to polymerase inhibitors remains to be established, particularly in patients with virologic failure to DAAs and/or advanced liver disease.

## Background

Among viral factors that may guide the treatment option the most important is the viral infecting genotype. The NS5b inhibitors are classified into nucleoside/nucleotide inhibitors (Ni) and non-nucleoside inhibitors (NNi). The Ni are considered active across all HCV genotypes, while NNi show restricted spectrum of activity against various genotypes, due to the rate of divergence of the binding site among different genotypes.

One other important viral factor is the genetic barrier to resistance related to the number and type of nucleotide substitutions required for the emergence of RASs during replication. The genetic barrier varies with drug class specificity over than HCV genotype and subtype [1].

In addition to the genetic barrier, the in vitro resistance level, corresponding to fold change under effective concentration (EC50) of a single compound in the replicon system, is commonly used to define the resistance profile of drug-selected variants [2–4]. The fitness of the variant under drug selection (replicative capacity of a resistant variant) may play also a crucial role in the virologic outcome [5].

Therefore, clinically relevant resistant strains are the result of a dynamic interaction between the fold change, the genetic barrier, and the replicative capacity of the virus selected during treatment with DAAs.

In this complex scenario, one important and debated possible virologic factor that may negatively influence the response to DAAs is the presence of RASs before any treatment with DAAs Baseline resistance testing is currently not routinely considered or recommended for initiating HCV treatment with DAAs, due to the overall high response (SVR) rates (> 90%) [6–8].

However, pre-existing RASs in clinical strains have been shown to influence the virologic outcome for the combination of protease inhibitor asunaprevir with NS5a inhibitor daclatasvir [6] or for the combination of elbasvir/grazoprevir [7], or in patients with cirrhosis prior to daclatasvir/sofosbuvir [8].

The studies on the impact of pre-existing NS5b RASs on the virologic outcome gave discordant results [9, 10].

For the NS3 protease gene, Pickett et al. [11] showed that HCV G1a isolates can be separated into at least two distinct clades, designated I and II. Several informative sites for this distinction are located within or proximal to codons associated with resistance to protease inhibitors or polymerase inhibitors. Santos and colleagues [12] showed that NS3 80 K fell into a specific sub clade of G1a cladeI: NS3 80 K polymorphism was associated with subclade IA, but nearly absent in subclades IB and IC. This finding providing an explanation for different resistance profiles to protease inhibitors of isolates clustering within cladeI.

This peculiar characteristic of HCV G1a suggests clade-specific differences that could modify the susceptibility of some clinical isolates to different classes of compounds.

A number of studies investigated the frequency of naturally occurring RASs into the NS5b domain of isolates retrieved from international data base [13, 14]. However, few data [15] are available on the presence of RASs within the NS5b domain according to geographic origin. Therefore, we decided to examine the frequency in isolates obtained from an international database, to better characterize genetic differences of HCV subtypes 1a and 1b according to their geographic origin and distribution within clades.

## Methods

The analysis considered only sequences of HCV G1a and G1b for whom was available the geographic origin. In total 1117 G1 NS5b sequences deposited before 2010 (to ensure that sequences were obtained from patients before exposure to DAAs activity) were obtained from Los Alamos HCV database, after exclusion of sequences containing stop codons in NS5b region, multiple sequences from the same patient and recombinant or clonal sequences. Of these 1117 isolates, 116 sequences were removed because G1 subtype was not indicated, 7 sequences clustering with G1 non-a non-b subtype were excluded, and 156 sequences for whom geographic origin was not available were also removed.

Thus, present study included 738 NS5b sequences: 432 clustered with G1a and 306 with G1b. Mutations analysis was performed at the resistance-associated positions for NS5b mutations already described in literature according to Ni or NNi compounds (Table 1).

**Table 1:** Summary of substitutions associated with resistance to nucleoside and non-nucleoside NS5B inhibitors [26–28]

Position	RAS	Genotype	Drug
159	F	1a	SOF
237	G	1a	SOF
282	R/ **T**	1a	SOF
316	F	1a	SOF
320	F	1a	SOF
321	A	1a	SOF
159	F	1b	SOF
282	G/T	1b	SOF
316	F/H/N	1b	SOF
321	I	1b	SOF
314	H	1a	DSV
316	**Y**	1a	DSV
414	**T/V**	1a	DSV
448	**C/H**	1a	DSV
553	**I** /V	1a	DSV
554	**S**	1a	DSV
556	G/N/R	1a	DSV
558	R	1a	DSV
559	G	1a	DSV
316	Y/ **H** /N	1b	DSV
368	T	1b	DSV
414	I	1b	DSV
445	F	1b	DSV
448	C	1b	DSV
553	V	1b	DSV
556	**G**	1b	DSV
559	G/N	1b	DSV
421	**V**	1a	BCV
495	**L/S**	1a	BCV
499	A/any	1b	DLV

*BCV* beclabuvir, *DSV* dasabuvir, *SOF* sofosbuvir, *DLV* deleobuvir

Amino acid substitutions detected in vivo in DAA failing patients are underlined, independently of in vitro data availability. Additionally, RAS detected only in vitro are indicated in bold

According to previous report [12], amino acid changes were considered as polymorphic when present in at least 10% of isolates and non-polymorphic when detected in < 10% sequences.

### Statistical analysis

Data were analyzed using Chi-Square or Fisher exact, when appropriate. A *P*-value < 0.05 was considered statistically significant.

## Results

### Genotype distribution according to geographical origin of isolates

The geographic distribution of genotype 1 according to subtypes, showed that of 432 G1a sequences analyzed 63 isolates were from Europe, 368 from USA and only 1 sequence was from Asia. Of 306 G1b isolates, 69 sequences were from Europe, 137 from USA and 100 from Asia. The comparison of isolates from USA and Europe showed that sequences from USA belonged more frequently to G1a (73% sequences) than to G1b (27% sequences), while G1a (48% sequences) and G1b (52% sequences) were equally distributed in Europe, *P* < 0.0001. Concerning the distribution of clades in G1a isolates, we found that clade I and II were differently distributed between isolates from USA and Europe. In isolates from USA, 280/368, (76%) sequences belonged to cladeI, and the remaining 88 sequences to cladeII (24%). In contrast, 19/63 (30%) sequences from Europe clustered with cladeI and 44/63 (70%) sequences with cladeII, *P* < 0.0001.

### Global distribution of RASs in genotype 1

In genotype 1, 28% of sequences were mutated in sites of resistance to NS5b by population analysis. HCV NS5b sequences clustering with HCV G1a were more conserved in regard of RASs than HCV G1b isolates, (60/432, 13.8% vs.148/306, 48.3%, mutated sequences, *P* < 0.0001).

### Geographic distribution of RASs and polymorphisms in genotype 1a

In total, for G1a, RASs were detected in 60/432 (14%) sequences. RASs were differently distributed between isolates from Europe (15/63, 24%) and USA (45/368, 12%) *P* = 0.0186. Only one isolate from Asia clustered with HCV genotype 1a and had not RASs.

**Fig. 1:**
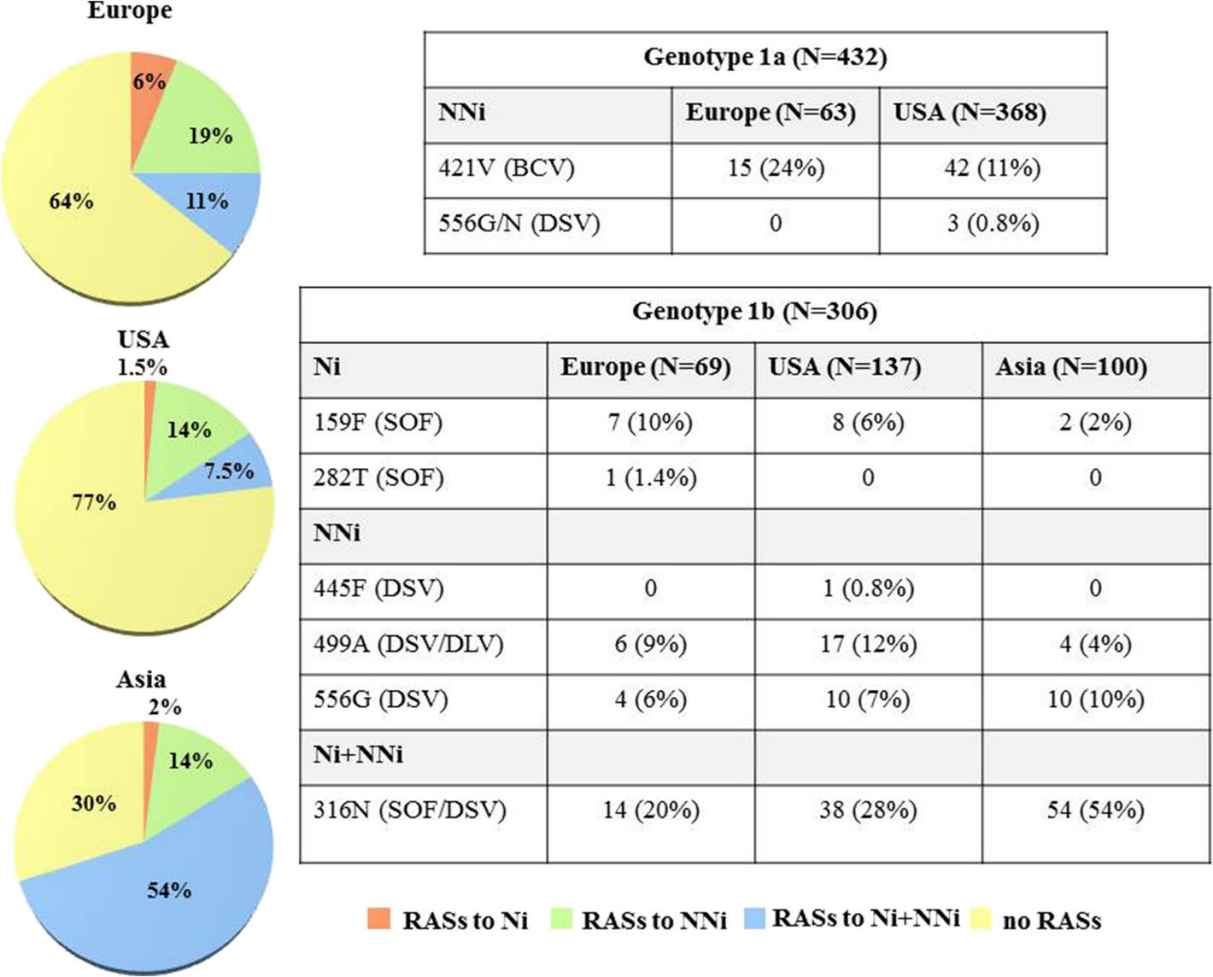
Frequency and geographic distribution of RASs to NS5b Ni or NNi. BCV = beclabuvir, DSV = dasabuvir, SOF = sofosbuvir, DLV = deleobuvir

Mutation 421 V with low level resistance (FC = 3) to NNi beclabuvir, was more frequently detected in isolates from Europe (15/63, 24%) than in isolates from USA (42/368, 11%) *P* = 0.0140. This aa change was polymorphic in Europe as well as in USA isolates (Fig. 1).

Despite this polymorphism was present in G1a cladeI as well as in G1a cladeII sequences from Europe and USA, a different distribution of RAS 421 V was observed in isolates belonging to G1a cladeII according to different geographic origin: this mutation was detected in 12/44 (27%) G1-cladeII isolates from Europe, and in 2/88 (2%) G1-cladeII isolates from USA, *P* < 0.0001. None of analyzed sequences had RASs to deleobuvir.

None of RASs described for sofosbuvir was detected, while 3 mutated sequences at position 556G/N (FC = 30) and associated with resistance to NNi dasabuvir were found, all among USA isolates. Fold change for each RAS and replicative capacity of G1a resistance mutations are depicted in Table 2.

**Table 2:** Fold change and replicative capacity of naturally occurring RASs in isolates retrieved from Los Alamos HCV database

RAS	DRUG^a^	HCV subtype	Fold change	Replicative capacity	References
421 V	BCV	1a	3	NA^b^	–
556G	DSV	1a	30	59%	[2]
556 N	DSV	1a	29	NA	–
282 T	SOF	1b	10	5%	[3, 4]
159F	SOF	1b	1.3	25%	[3, 4]
316 N	SOF	1b	1.6	154%	[2]
	DSV		5	154%	[2]
556G	DSV	1b	11	62%	[2]
445F	DSV	1b	16	NA	–
499A	DLV	1b	6	NA	–
	DSV	1b	1.4	NA	–

^a^*BCV* beclabuvir, *DSV* dasabuvir, *SOF* sofosbuvir, *DLV* deleobuvir ^b^*NA* not available

### Geographic distribution of RASs and polymorphisms in genotype 1b

A high prevalence of NS5b RASs was detected in G1b (176/306; 57% sequences were mutated at sites associated with resistance). Considering all sequences clustering with G1b, a different distribution of RASs according to geographic origin, was found. RASs were detected in 32/69 (45%) sequences from Europe, in 74/137 (54%) isolates from USA and in 70/100 (70%) sequences from Asia (*P* = 0.0051). The distribution of RASs to Ni vs NNi according to geographic origin is described in Fig. 1.

In detail, the 159F (FC = 1.3 to Ni sofosbuvir) was present in 7/69 (10.1%) European sequences, in 8/137 (5.8%) sequences from USA and in 2/100 (2%) Asian sequences, showing a trend toward significance, *P* = 0.0743 (Fig. 1).

The RAS 316 N, (FC = 1.6 to Ni sofosbuvir, and FC = 5 to NNi dasabuvir) was more frequently detected in Asian isolates (54/100, 54%) respect to isolates from USA (38/136; 28%) and Europe (14/69; 20%), *P* < 0.0001. The RAS 316 N was polymorphic in Asia as well as in Europe and USA, being present in 54% Asian isolates and in > 10% of sequences from Europe or USA (Fig. 1). The RASs 159F and 316 N were concomitantly detected in 2% isolates from Asia, 5% of isolates from USA and 10% isolates from Europe.

Twenty-seven/306 (8.8%) sequences harbored the RAS 499A (FC = 6 to NNi deleobuvir; FC = 1.4 to NNi dasabuvir): 17/137 (12.4%), were from USA, 6/69 (8.6%) from Europe and 4/100 (4%) from Asia, this result showing a trend towards significance, *P* = 0.0788. So, on the basis of data retrieved from Los Alamos, the 499A substitution seemed polymorphic in USA but not in Europe and Asia. The 556G substitution (FC = 11 to dasabuvir) was similarly distributed among isolates from Asia, USA, and Europe, being detected in 10% (10/100) of Asian isolates, 7% (10/137) of isolates from USA, 6% (4/69) of European sequences, *P* = 0.577 (Fig. 1). One sequence from Europe had 282 T (FC = 10 to Ni sofosbuvir) and 1 sequence from USA had 445F (FC = 16 to dasabuvir). Finally, the 316 N associated with 556G was detected in 4/137 (3%) isolates from USA, in 3/69 (4%) isolates from Europe and in 3 (3%) sequences from Asia. Fold change of each RAS and replicative capacity of G1b resistance mutations are shown in Table 2.

## Discussion

In the present study, we observed a higher prevalence of RASs in G1b respect to G1a and a geographical distribution of RASs as well as polymorphic aa change in G1a and G1b, by retrieving sequences from an international data base, Concerning G1a sequence analysis, we showed that the aa substitution 421 V was polymorphic (detected in > 10% of sequences) in G1a isolates from USA and Europe.

In the study by Patino–Galindo et al. [13], evaluating global prevalence of natural RASs to polymerase inhibitors, 421 V was detected in 12.56% of G1a sequences; however, the geographic origin of polymorphisms was not reported.

Recently, some reports [12, 16, 17], investigated the geographic distribution of polymorphism NS3 80 K and its segregation into a specific clade, providing explanation for different resistance profiles to protease inhibitors of isolates from different geographical area and within different clades. Interestingly, we found that the isolates from Europe harboring NS5b 421 V polymorphism fell more frequently in G1a cladeII, while 421 V polymorphism in USA clustered more frequently within G1a cladeI. The majority of 421 V G1a cladeII mutated sequences detected in Europe were from Switzerland. Therefore, it is possible that this polymorphic site within G1a cladeII is a peculiar characteristic of isolates from this country rather than a common characteristic of European strains.

Unfortunately, in the present study no additional sequences from other countries in Europe were available to draw conclusions on the distribution of 421 V polymorphism into different clades (I or II) according to geographical origin of isolates.

In line with previous studies [13, 15], mutation 556G (RAS to NNi dasabuvir but not to NNi deleobuvir and Ni sofosbuvir) was rarely detected in G1a (0.6% of isolates).

Concerning the G1b isolates, we globally detected 5% of 159F substitution. By population analysis, the 159F was polymorphic in Europe, but not in Asia and USA. This data is in accordance with that of Patino–Galindo et al. [13] and Welzel et al. [15], indicating that the 159F was present respectively in 4.5% or 8% of isolates obtained from an international database .

In our study, the frequency of 159F was similar to that described in a previous study [4], exploring the baseline resistance profile of isolates obtained from sofosbuvir (SOF) or ledipasvir (LDV) plus sof trials and showing that the 159F was present by deep sequencing cut off 15%, in 7% G1b patients. In this report was also investigated the presence of baseline RASs for polymerase inhibitors in individuals with virologic failure, showing that 2/6 patients harboring the mutation 159F in sof trials and 0/23 in SOF/LDV trials, experienced virologic failure. The authors concluded that baseline 159F was not associated with treatment failure because the rate of treatment response in these two studies were similar in individuals with or without 159F.

In another study, Isakov et al. [18], showed that, among G1b infected patients, the presence of 159F at baseline was associated with a lower SVR rate in patients treated for 16 weeks, respect to those treated for 24 weeks.

In liver pre-transplant studies [18], 4/4 G1b infected patients with pretreatment 159F as dominant variant (> 99% within the viral population) experienced virologic failure. Considering aforementioned studies, it is possible that not only the duration of the regimen including a single DAA and/or the presence of advanced liver disease, but also the proportion of quasispecies harboring 159F polymorphism at baseline, have contributed to the failure. In the majority of reports [9, 14, 20] evaluating natural RASs to polymerase inhibitors, the 316 N associated with resistance to dasabuvir and sofosbuvir was detected in 10–36% of G1b isolates. We found the aa substitution 316 N in 35% of G1b sequences, with higher frequency in Asian isolates compared to those from Europe or USA.

In contrast, the study by Chen et al. [19] showed that, none of 361 sequence of genotype 1b harbored 316 N, despite isolates from Asia were also included in their analysis. According to previous studies performed by means of population sequencing in clinical strains from Asia, that did not found [21], or found at very low percentage [22] the 159F associated with 316 N, these two mutations were concomitantly present in only two isolates coming from Japan, despite the high frequency of 316 N in this area. However, in the study by Ito et al. [22], the deep sequencing analysis of clinical strains demonstrated a high frequency (30%) of 159F among patients harboring 316 N polymorphism. In the present study, we showed that a high number of isolates in Europe and USA naturally harbored 316 N in combination with 159F. In this regard, the aa substitution 316 N has been described as a compensatory mutation after the selection of the 159F mutation, giving a selective advantage of the variant with concomitant 159F and 316 N. In vitro studies [3, 4] showed that the replication capacity of 159F is relatively low, 0.24–0.25 fold compared with the wild-type virus, while in the case with the co-existence of the 159F and the 316 N substitutions, the replication capacity is 0.65–1.14 fold respect to the wild-type virus. Therefore, the higher replication capacity of the double mutant respect to the 159F variant and to the wild type, may confer a higher level of resistance than 159F alone to Ni sofosbuvir. Unfortunately, the presence of this double mutant was not evaluated in the study by Welzel et al. [15], exploring a large number of sequences around the world and coming from clinical trials. So, no additional information on its frequency in global population is available so far.

In this context, Donaldson et al. [23], using bioinformatics analysis, showed that the cysteine to asparagine change at NS5b position 361 introduces a larger aa than the C316 and that the larger amino acid is predicted to interfere with the ability of sofosbuvir to enter the active site, providing molecular basis for its potential role in the resistance pattern to Ni sofosbuvir.

In G1b, RAS 499A (RAS to NNi deleobuvir and dasabuvir) was polymorphic in USA but not in Europe and Asia. This RAS was previously detected in 16.8% of patients from China [21]. In one other study [13] 499A was detected in 9.8% of G 1b isolates. However no information was provided on the geographic origin of this RAS.

According to previous report [13], the S556G was more frequently detected in G1b respect to G1a (7% vs. 0.6% of frequency, respectively). One study from China [22] showed 3.7% of 556G/N in G1b infected patients. In two other studies [9, 19] 556 N was present in 9.6% and 7% 1b isolates, respectively and in 1.4% and 0.6% 1a isolates, respectively.

In one recent clinical trial [24] evaluating the efficacy of 8 weeks treatment with ombitasvir /paritaprevir /ritonavir/dasabuvir, in G1b infected non cirrhotic patients, 2 patients who experienced relapse, harbored NS5b 316 N and NS5b 556G at baseline and at the time of relapse. In such cases natural polymorphisms in sites of resistance might affect treatment outcome. In regard of this issue, one clinical study [25] of re-treatment with polymerase inhibitors-containing regimens, showed that 159F associated or not with 282 T was more frequently selected when patients previously exposed to SOF/LDV for 8 or 12 weeks, were re-treated with the same regimen for 24 weeks (2/12 virologic failure among 41 re-treated patients, had 159F alone or associated with 282 T).

Profiling of geographic distribution for polymorphic aa changes could add important information for the future treatment strategies; the mutational profile of NS5b polymerase region according to different HCV subtypes and their geographic origin, can be important especially in difficult-to-cure patients for whom new treatment strategies could involve complex association regimens. Viruses carrying polymorphisms and in particular those harboring two or more RASs may attenuate Ni and NNi activity affecting future treatment options.

## Abbreviations

aa: Amino acid
BCV: Beclabuvir
DAAs: Direct acting antivirals
DLV: Deleobuvir
DSV: Dasabuvir
EC50: Effective concentration 50
FC: Fold change
G: Genotype
HCV: Hepatitis C virus
LDV: Ledipasvir
Ni: Nucleoside/ nucleotide inhibitor
NNi: Non-nucleoside inhibitor
RAS: Resistance associated substitutions
SOF: Sofosbuvir
